# Impact of the Stopcut project on the practice of Female Genital Mutilation/Cutting in Southwest Nigeria: a quasi-experimental study

**DOI:** 10.1186/s12889-025-21976-1

**Published:** 2025-02-24

**Authors:** Oluwatomi Olunuga, Rhoda Robinson, Paul Ojajuni, Winnie Opondo, Wendyjoy Gitari, Isaiah Owolabi, Jonathan Izudi, Boscow Okumu

**Affiliations:** 1 HaCEY Health Initiative, Lagos, Nigeria; 2 African Population and Health Research Centre, Manga Close, Off Kirawa Road, P. O. Box 10787-00100, Nairobi, Kenya; 3 Environment for Development (EfD) Centre, School of Economics, University of Nairobi, Nairobi, Kenya

**Keywords:** Female genital mutilation, Interventions, StopCut

## Abstract

**Background:**

Female Genital Mutilation/Cutting (FGM/C) is a harmful practice recognized as a gross violation of human rights and violence against women. Nigeria has been identified to share an overwhelming amount of burden as regards FGM/C. Despite several interventions having been implemented to tackle the practice of FGM/C, progress has been slow, and the results are generally mixed coupled with weak law enforcement, inadequate public awareness, and deeply rooted cultural beliefs. In response to these challenges, the StopCut project was launched to protect women and girls from FGM/C in Southwest Nigeria. This study examines the impact of the StopCut project on the practice of FGM/C in the region.

**Methods:**

To measure the impact of the StopCut project, we used a quasi-experimental design specifically the Inverse Probability Weighting approach. A multistage sampling approach was used to collect data from 413 households across 12 Local Government Areas (LGAs) in Nigeria. A well-structured interviewer-administered questionnaire was used to collect information on knowledge, attitudes, and practices related to FGM/C, and respondents’ attitudes towards reporting and continuing FGM/C.

**Results:**

the study revealed that the StopCut project had significant positive impacts on knowledge and behaviour related to FGM/C. Specifically, the study revealed that participation in the project significantly increased knowledge of FGM/C consequences, awareness of the Violence Against Persons Prohibition (VAPP) Act, willingness to report FGM/C incidents, and reporting of FGM/C practices within families.

**Conclusion:**

The findings indicate that the StopCut intervention effectively improved participants’ knowledge about FGM/C, legal frameworks, and reporting behaviour. The study thus highlights the need for a more comprehensive approach to challenging cultural beliefs and scaling up the interventions to other areas as well.

**Supplementary Information:**

The online version contains supplementary material available at 10.1186/s12889-025-21976-1.

## Introduction

Female Genital Mutilation/Cutting (FGM/C) is a common age-long practice across the globe. It is defined by the World Health Organization (WHO) as the partial or complete removal of the external female genitalia for non-medical purposes [1]. It is a practice that is performed primarily for traditional and cultural reasons on women and girls without informed consent of its consequences. This practice has gained the attention of various groups of people worldwide as different health implications are beginning to result from FGM/C [2]. It is a violation of the basic human rights of the individual who is made to undergo this practice as it is done against the person’s wish in most cases [3].

There are four major classifications for FGM/C by the WHO of 1997 [4]. They include Type I (clitoridectomy), Type II (excision), Type III (infibulation), and Type IV which includes other harmful practices to the female genitalia for non-medical purposes [5]. Type I is the partial or total removal of the clitoris and in some cases, only the prepuce. Type II is the partial or total removal of the labia minora and clitoris and can be accompanied by the excision of the labia majora in some cases. Type III involves the narrowing of the vaginal opening by repositioning and cutting the inner or outer labia with or without the removal of the clitoris. The cut areas are stitched for a period to have a covering seal and a small opening left for urine and menstruation. This type of FGM/C is considered the worst of all the types. Type IV includes practices such as burning the clitoris, stretching the labia or clitoris, piercing, pricking, or incising the clitoris, or the introduction of harmful substances into the vagina to cause a narrowing of the vaginal opening [5].

In the South West of Nigeria, the prevalent types of FGM/C are Type I and Type II, which align with traditional and cultural practices aimed at controlling female sexuality and ensuring marital fidelity [6]. These practices are common across the three respondent states—Oyo, Osun, and Ekiti—which share cultural similarities such as patriarchal societal structures, deeply rooted traditional beliefs, and the significant role of elder women in perpetuating these practices. Gender practices, such as assigning domestic roles predominantly to women and leadership roles to men, further reinforce norms that promote FGM/C [7]. Understanding these socio-cultural and economic settings is crucial for identifying the underlying norms that sustain the practice and for developing socially relevant, multifaceted approaches to address the problem from its roots.

In contrast to male circumcision, there are no known health benefits of FGM/C nor is it performed for medical reasons [8]However, it is recognized to cause severe short- and long-term consequences to both physical and psychological health. Some of the complications include developing clitoral cysts, bleeding, fistula, obstetric issues, urinary infections and retention, vaginal tears, and psychological trauma [2, 9–12].

Nigeria has been identified to share an overwhelming amount of burden as regards FGM/C. The Thomson Reuters Foundation [13] reports an FGM/C prevalence of 24.8% among women of reproductive age in Nigeria. Furthermore, about 20 million women and girls have been mutilated or cut, which represents 10% of the global total. The practice has mainly been associated with a mix of cultural, religious, and social factors families, and communities [14]. In some cultures, FGM/C is an important step towards raising a girl child and preparing her for marriage anchored on perceived appropriate sexual behaviour that links FGM/C to premarital virginity and marital fidelity. It is also believed that FGM/C reduces a woman’s libido and is expected to discourage sexual promiscuity [3].FGM/C practice is widespread across the major ethnic groups in Nigeria and is largely carried out in Southwest Nigeria by traditional circumcisers, known in Yoruba culture as ‘Oloola’ or in some cases health workers including medical doctors and nurses [15]. Most of the local cases are often carried out in unhealthy conditions, and without proper medical care and supervision, thus leading to serious health complications [11, 16].

Over the years numerous studies have emerged to assess the effectiveness of various interventions toward ending FGM/C in sub-Saharan Africa [9, 10, 15–27].

Most of the studies are mainly descriptive qualitative case studies and scoping reviews [25, 17, 26, 12, 18, 20, 24, 27, 28, 29], with very few empirical studies [19, 22, 23, 30]. In addition, there are relatively few studies that have looked at changes in knowledge attitudes and practices in Nigeria [15, 18, 25, 27]. An overview of existing literature reveals significant differences in applied definition, contextual factors, and methodological approaches making comparison of respective results difficult, creating demand for more current and relevant inquiry.

Moreover, despite the numerous interventions towards ending FGM in Nigeria such as the StopCut project, context-specific evidence, especially from an area with a high prevalence of FGM/C largely anecdotal to inform policy interventions is lacking. It is also inherent to identify which components of the Stop Cut project are effective and which are not. It is against this backdrop that the study aims to assess the impact of the StopCut project on community members’ knowledge, attitudes, beliefs, and practices regarding FGM/C. Specifically, the study seeks to: (i) Determine the impact of the project on knowledge of the consequences of FGM/C; (ii) Determine the extent to which the project increased community members’ knowledge of the position of the law against FGM/C; (iii) Determine the impact of the project on the willingness to report suspected and known FGM/C cases within the target community through formal channels or informal community-based mechanisms; (iv) Determine the impact of the project on the continued practice of FGM/C within households and among girls/women in the target community.

The findings of this study will be crucial in identifying successful strategies and highlighting areas where further action is needed to protect the rights and well-being of women and girls. The study findings will inform policy and strengthen the synergy between evidence generation and FGM/C interventions while contributing to the body of evidence. The findings will help us understand the project’s overall effectiveness towards guiding the design of scalable and adaptable interventions that can be implemented by other organizations working to eradicate FGM/C in Nigeria and beyond while informing policy advocacy.

## Setting the context

In recent decades, there has been a rise in the efforts to eliminate FGM/C practice in Nigeria within legal frameworks. In 2015, the Violence Against Persons Prohibition (VAPP) Act was passed in Nigeria which includes provisions for the criminalization of FGM/C [27].The National Policy and Plan of Action for the Elimination of FGM in Nigeria (2021–2025) also seeks to eliminate the practice of FGM/C in Nigeria to improve the health and quality of life of girls and women. Some States have laws and policies against FGM/C, however, for some reason, citizens are unaware of these Acts or laws [25] while the enforcement of FGM laws is reportedly low. The system for reporting harmful practices, either intended or unintended, is uncoordinated, causing citizens distrust to the system [31]. Despite the efforts of stakeholders to end FGM/C practices in Nigeria, the prevalence remains high due to factors such as lack of coordinating agencies, inadequate systems for law enforcement, lack of public awareness of existing policies and laws, poor reporting of FGM/C cases, low capacity of existing anti-FGM groups and civil society organizations (CSOs) to advocate for policy implementations and enforcements in Nigeria [13, 31, 32]. Further, deeply ingrained cultural beliefs and social norms continue to perpetuate the practice, and the enforcement of existing laws remains inadequate. Furthermore, the COVID-19 pandemic contributed to the increase in FGM/C practice, with lockdowns and disruptions to services creating a conducive environment for the secret practice of FGM/C [33].Lockdowns and school closures during COVID-19 confined girls to their homes, increasing their vulnerability to FGM/C as families exploit the privacy afforded by these restrictions to perform the practice undetected [38]. Additionally, the diversion of healthcare resources to combat the pandemic has led to the suspension of anti-FGM/C programs and reduced access to protective services, leaving at-risk individuals without crucial support [34].

This necessitated the need for continued community education, awareness, and advocacy at the community and policy formulation level to ensure that negative behavioural patterns and the myths and beliefs that strengthen the practice are adequately addressed and resolved. It is against this background that the Stop Cut project came into force. The Stop Cut project, a three-year intervention supported by the United Nations Trust Fund to End Violence Against Women, represents a significant step in this direction. Implemented in 72 communities across Ekiti, Oyo, and Osun states in Southwest Nigeria, the project aimed to protect women and girls from FGM/C by improving policy and law implementation. The Stop Cut project employed a multi-pronged strategy, combining community education and sensitization, research, capacity building, and advocacy as presented in the program theory of change in the Annex. Specifically, the project comprised of the following key activities:

(i) **Establishment of an EndFGM Alliance**: This comprised representatives from the government, CSOs, media agencies, community leaders, and law enforcement officers. The alliance members collaborated to implement advocacy and campaign activities and participated in learning and sharing key challenges and strategies to address FGM/C applicable in local contexts.
(ii) **Capacity-building workshops for stakeholders**: This was targeted at government officials, CSOs, media professionals, and community and religious leaders. The workshops aimed to increase the knowledge of the participants on FGM/C and its health consequences, the laws and policies that prohibit FGM/C, and strategies for increasing the advocacy strength at the grassroots to eradicate FGM/C practice.
(iii) **Community sensitization campaigns**: These were held within the community and targeted women and girls including survivors, men and boys, community leaders, and circumcisers living in the project communities to increase their knowledge about the health consequences of FGM/C, the laws, and policies that prohibit it, and the reporting channels and referral mechanism for survivors. The outreaches and campaigns were held in schools, worship centres, market squares, town halls, and among local groups in the communities.
(iv) **Advocacy visits to policymakers**: The project teams comprising government workers, media, and CSOs visited policymakers including governors, the governor’s wives, lawmakers (State Senate and House of Representatives), and traditional leaders among others. The meetings aimed to present a case for the review of negative cultural practices that marginalize women and girls such as FGM/C and the strengthening of state laws to protect women and girls from FGM/C.
(v) **Media campaigns against FGM/C**: This includes social media community engagement (Twitter live and Instagram live events), virtual conferences on ending FGM/C, radio and television public service announcements (PSAs) and jingles, radio and TV programs including NEWS coverage, and multimedia content targeted at increasing the public knowledge about FGM/C, provisions of the VAPP Act 2015 and state laws on FGM/C, and leveraging media to engage young people to advocate for the abandonment of FGM/C practice in their communities.
(vi) **Stakeholder engagements**: This was done through focus groups, debrief meetings, and consultation forums with state and non-state actors across the project states to secure their buy-in and ensure that the project strategy is improved and repositioned to achieve the set outcomes and goal. In these meetings, project reports were presented and disseminated to inform further programmatic actions and advocacy.

These interventions were expected to lead to the following outcomes: improved knowledge, reporting, and enforcement of FGM/C policies in project states; positive behaviour change among citizens in project states regarding ending FGM/C practices; and CSOs, media, government, and citizen use of evidence-based data and information on prevalence, contribution factors and level of policy implementation to support efforts in ending FGM/C in project states. This would finally lead to reduced prevalence of FGM/C and protection of women and girls from FGM/C.

While the StopCut project has made significant strides in raising awareness and mobilizing communities against FGM/C, the practice keeps evolving through social norms, medicalization^1^, myths, and other factors which still promote its continuation. FGM/C is deeply rooted in cultural traditions and beliefs, often seen as a rite of passage to maturity for women and upholding notions of purity, virginity, fertility, control of immorality, and marriageability. These entrenched social norms and myths continue to legitimize FGM/C, despite its proven negative health consequences, gender-based violence, and human rights violations. Further, despite the existence of laws prohibiting FGM/C such as the Violence Against Persons Prohibition Act in 2015, which criminalizes the practice enforcement of these laws remains a challenge with many states yet to fully adopt the legislation, leading to a lack of accountability and an environment where FGM/C can still thrive. From our engagement with NGOs and CSOs working at the community level, it was reported that many cases of FGM/C are likely unreported due to the culturally sensitive nature of the practice, as victims and their families may be reluctant to come forward or seek help. This lack of reporting may hinder effective monitoring, intervention, and enforcement efforts by the government and CSOs, perpetuating the practice. Additionally, weak coordination and collaboration among stakeholders undermine efforts to address FGM/C effectively in Nigeria. It’s therefore inherent to take stock of the steps made so far and identify whether the interventions are effective, as this will help identify critical areas for further action to inform policy.

## Methodology

### Study design

To quantify the impact of the StopCut project, the study used a quasi-experimental approach specifically the Inverse Probability Weighting (IPW) approach. The main interest of the study is the average treatment effect on the treated (ATT). That is, how participating in the StopCut project changes knowledge, attitude and practice of FGM/C. Since it is not possible to observe what the results would have been in the absence of the StopCut project, to handle the missing data on counterfactual, we identified households, that did not receive or get exposed to the project. Since the participation in the project is non-random, there is a high possibility of selection bias hence the reason for employing the IPW model to measure the average impact on knowledge and practices. The reporting of the study findings adhered to the Strengthening of the Reporting of Observational Studies in Epidemiology (STROBE) guidelines [35].

### Setting

This study was conducted in Oyo, Osun, and Ekiti in the South-Western region of Nigeria. The South-Western region is made up of 6 major states, out of which the study locations were selected. Oyo State, with its capital in Ibadan, is an inland state in southwestern Nigeria. It is bounded by Kwara State to the north, Osun State to the east, Ogun State to the south, and the Republic of Benin to the west. Covering an area of 28,454 square kilometers. The state has one teaching hospital and other healthcare facilities located in the area. The 2006 census recorded a population of 5,580,894 in Oyo. Agriculture is a major economic activity in the state and there are both urban and rural communities in the states.

Ekiti State is located in the Southwestern region of Nigeria. It was created on October 1, 1996, and initially had 16 local government areas (LGAs), which has since increased to 20. According to the 2006 census, Ekiti had a population of 2,210,957 and was projected to reach 3,270,800 by 2016. The people of Ekiti are culturally homogeneous and speak the Yoruba dialect. The town is mostly populated by individuals living in rural communities. Osun State was carved out of the former Oyo State in 1991, spanning an area of 14,875 square kilometers. Osun is bounded by Oyo State to the west, Ondo and Ekiti States to the east, Kwara State to the north, and Ogun State to the south. With a 2006 population of 3,416,959, Osun’s inhabitants are primarily Yoruba farmers, traders, and civil servants. The state has two teaching hospitals and numerous other healthcare facilities.

### Sampling method

A multistage sampling technique was used to select participants as follows:

Stage 1: Three project states (Oyo, Osun, and Ekiti) were purposively selected.

Stage 2: From each state, three intervention Local Government Areas (LGAs) and one control LGA (no intervention) were randomly selected using an Excel-generated random number list, resulting in 12 LGAs.

Stage 3: One ward was randomly selected from each LGA, resulting in a total of 12 wards.

Stage 4: Within each ward, 60 households with females were listed, and 34 households were randomly selected for the study.

Stage 5: Female respondents were randomly chosen from household member lists, resulting in 413 participants, determined using the Yaro formula.

### Data collection

Data was collected using an interviewer-administered questionnaire attached in the annex. The questionnaire collected data on respondent sociodemographic characteristics, knowledge of FGM, knowledge of FGM laws, intention to report FGM, practice of FGM, and intention to continue FGM practice. The questionnaire was developed based on a review of the existing literature on FGM and in consultation with subject matter experts. It was pilot-tested and refined before being deployed for full-scale data collection. The use of an interviewer-administered format allowed the research team to ensure completeness of responses, clarify any ambiguities, and provide additional context or explanations as needed. This approach helped to enhance the quality and reliability of the data collected.

Data were collected within two weeks from 9th March to 22nd March 2023. Twelve research assistants (male and female) with at least a university degree and a health background were trained in each state to collect the data. The training covered the data collection process using Kobo Collect, sampling technique, informed consent, ethics and safeguarding, and confidentiality. The selected research assistants were fluent in the local Yoruba language. This allowed them to effectively communicate with and interview respondents in their native tongue.

### Study variables

The study considered several key outcome variables namely: knowledge of Consequences of FGM/C i.e. whether the respondent was aware of the health consequences and complications associated with FGM/C; knowledge of FGM/C as a criminal offense that is the respondent’s awareness of the legal status of FGM/C as a criminal act according to existing laws; knowledge of the VAPP Act i.e. knowledge that the Violence Against Persons Prohibition (VAPP) Act contains provisions specifically prohibiting the practice of FGM/C; willingness to Report FGM/C; Perception that reporting FGM/C is wrong to assess whether respondents believed that reporting FGM/C cases performed on family members was morally or socially unacceptable; experience of FGM/C to assess whether her female respondent had personally undergone FGM/C; and whether respondents had the intention to practice FGM/C. The other explanatory variables collected were: the respondent’s age, gender, education status, marital status, number of children, and religion among others presented in Table A1 in the annex. The treatment in our study was therefore defined as self-reported participation in the StopCut project. Respondents were considered to have been exposed to the StopCut project if they reported that they had participated in at least two of the community activities of the StopCut project.

### Data analysis

The data were analyzed using Stata version 18, employing a combination of descriptive and econometric frameworks to ensure robust and reliable findings.

#### Descriptive analysis

Demographic Profiles: Descriptive statistics were used to summarize the sociodemographic characteristics of the respondents (e.g., age, gender, education, marital status, and religion). Outcome variables: Frequencies and percentages were calculated for key variables such as knowledge of FGM/C, awareness of laws, intention to report FGM/C, and intention to practice FGM/C.

#### Analytical framework

The study adopted a quasi-experimental econometric framework to estimate the Average Treatment Effect on the Treated (ATT). This framework assumes that the distribution of outcomes is independent of treatment (participation in StopCut) given observed covariates. The study used inverse probability of treatment weighting (IPTW) to create a control group that was similar to intervention group. The weighting created a control group of those who did not participate in StopCut that is as similar as possible to those who participated in StopCut. We weighed the control group using the negative inverse of the propensity scores (1/ [1-propensity score]) and the intervention group using the inverse of the propensity score (1/propensity score) as shown in previous studies [36, 37].The inverse probability weights mimic the matching intuition through reweighting to make the treatment and control group distribution look as similar as possible. However, identification of the average effect of the treatment within this framework requires both strict ignorability of treatment and the propensity score overlap [38, 39]. Another assumption is the common support in which similar individuals have a positive probability of being both project participants and non-project participants [40].The IPW regression model where the probability is derived from a logit model in line with the propensity scores is specified as:$\:\:Prob\:\left({X}_{i}\right)=\varLambda\:\left(X\varGamma\:\right)$

Sociodemographics and other covariates were included in the model. The IPW regression model was applied, where the propensity scores were used to reweight the data. In the model, propensity scores are first estimated to create the weights and define overlaps between comparison and control groups and then the weighted regression is estimated [41, 42].

The estimated model is a standard treatment effects regression, wherein the outcome variable of interest is regressed on the treatment together with controls from the propensity score regression [38–42]. This is done to control for any lingering covariate imbalance that could influence the estimates.

### Ethical consideration

The research team members were trained on the ethical process of data collection and obtaining informed consent. Informed consent was obtained from project participants. For participants under 18 years of age, consent was obtained from both the participants and their parents/guardians before data was collected. Interviews were conducted privately in secured locations to ensure the privacy of participants and the confidentiality of the information given. Data was securely stored away and restricted with a password to ensure that only authorized personnel have access to it for reporting purposes. Approval was obtained from the Ethical Review Committee of the Oyo State Ministry of Health, Osun State Ministry of Health, and Ekiti State Ministry of Health in Nigeria.

## Results

### Descriptive statistics

The summary statistics of the variables used in the study are presented in Table A1 in the annex. The results show that over half (51.1%) of the total sample were female, 54% were Christians, and 44% were married. The average age of the respondents was 30 years, and 81% had an education level beyond primary school. A detailed description of the other variables by treatment and control group is also presented.

### Knowledge, perceptions, and attitudes towards FGM/C

**Fig. 1:**
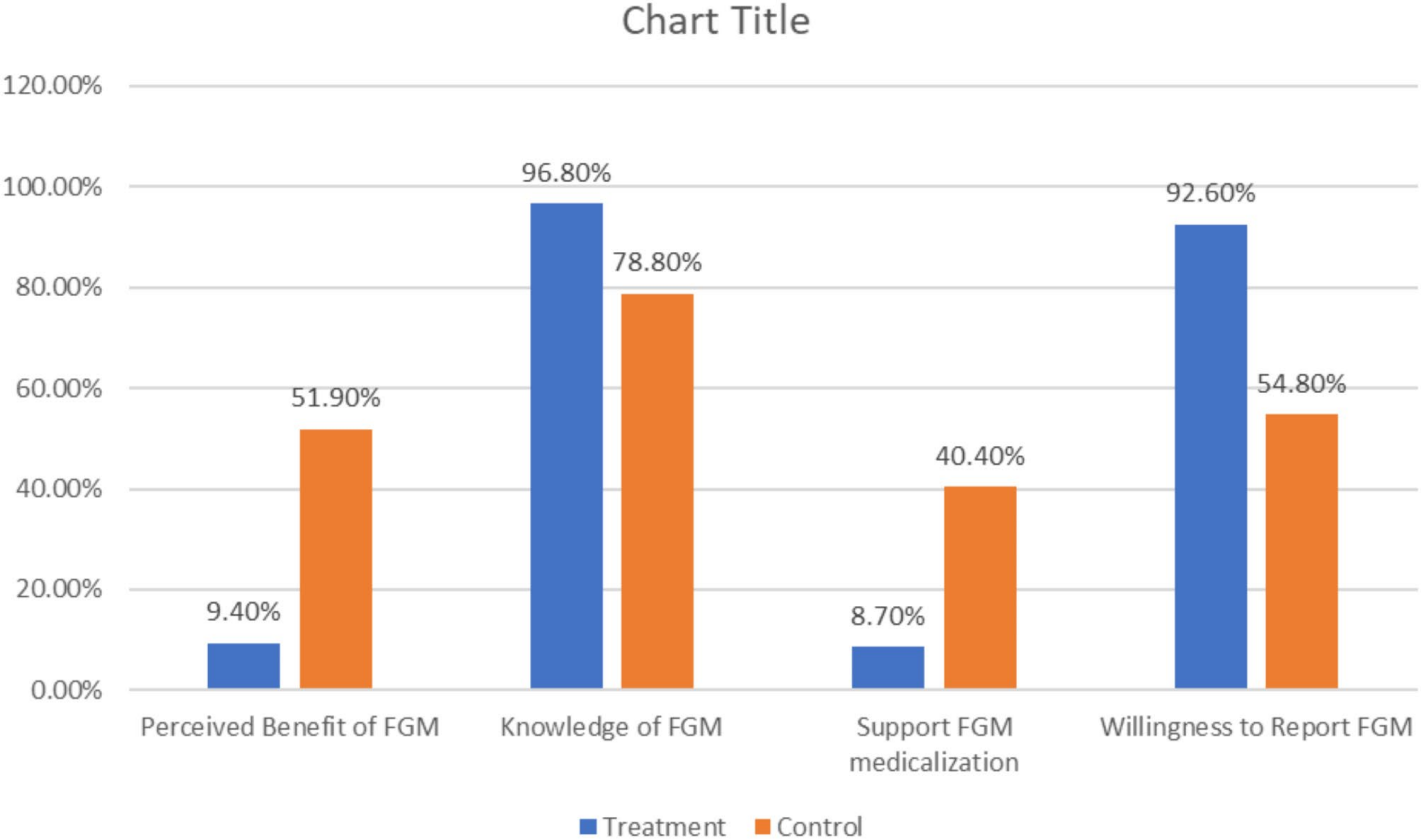
Knowledge attitudes and perceptions towards FGM/C

An examination of the perceptions of FGM/C revealed that 52% of the control group and 9.4% of the treatment group perceive FGM/C to be beneficial. Nearly all (92.6%) of the respondents in the intervention group were willing to report FGM/C, compared to 54.8% in the control group. Additionally, a small percentage (8.7%) of the treatment group supported the medicalization of FGM/C, whereas 40.4% of the control group also supported it. Further, 96.80% of the intervention group knew of FGM/C consequences (Fig. 1).

### Source of information on FGM/C

**Fig. 2:**
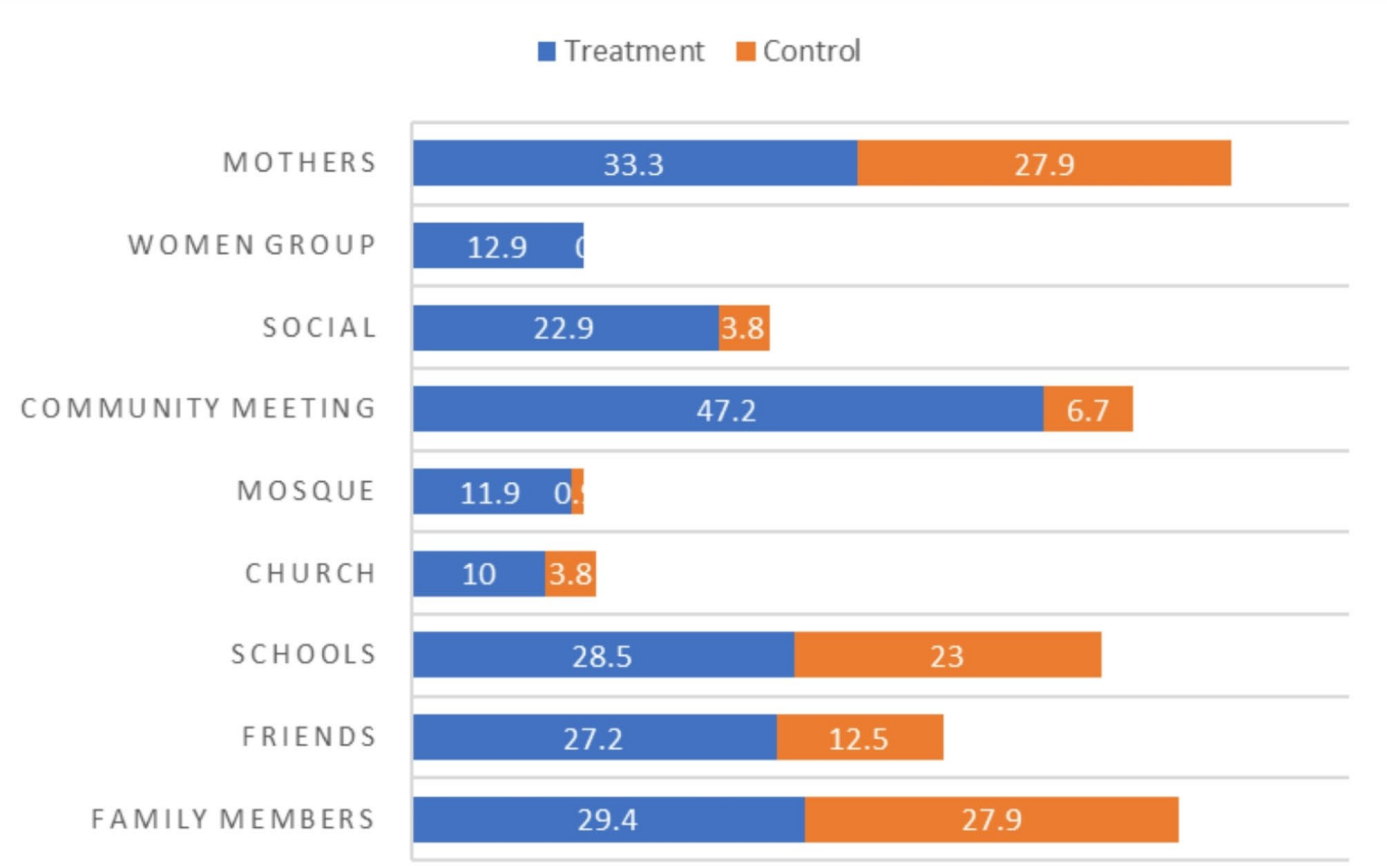
Source of information on FGM/C

Figure 2 presents information sources on FGM/C for the treatment and control groups. Among the treatment respondents, 47.2% heard about FGM/C from community meetings, 33.3% from their mothers, 29.4% from family members, 28.5% from schools, and 22.9% from social media. In contrast, within the control group, 27.9% heard about FGM/C from their mothers and family members, 23% from schools, and 12.5% from friends.

### Knowledge of consequences of FGM/C

**Fig. 3:**
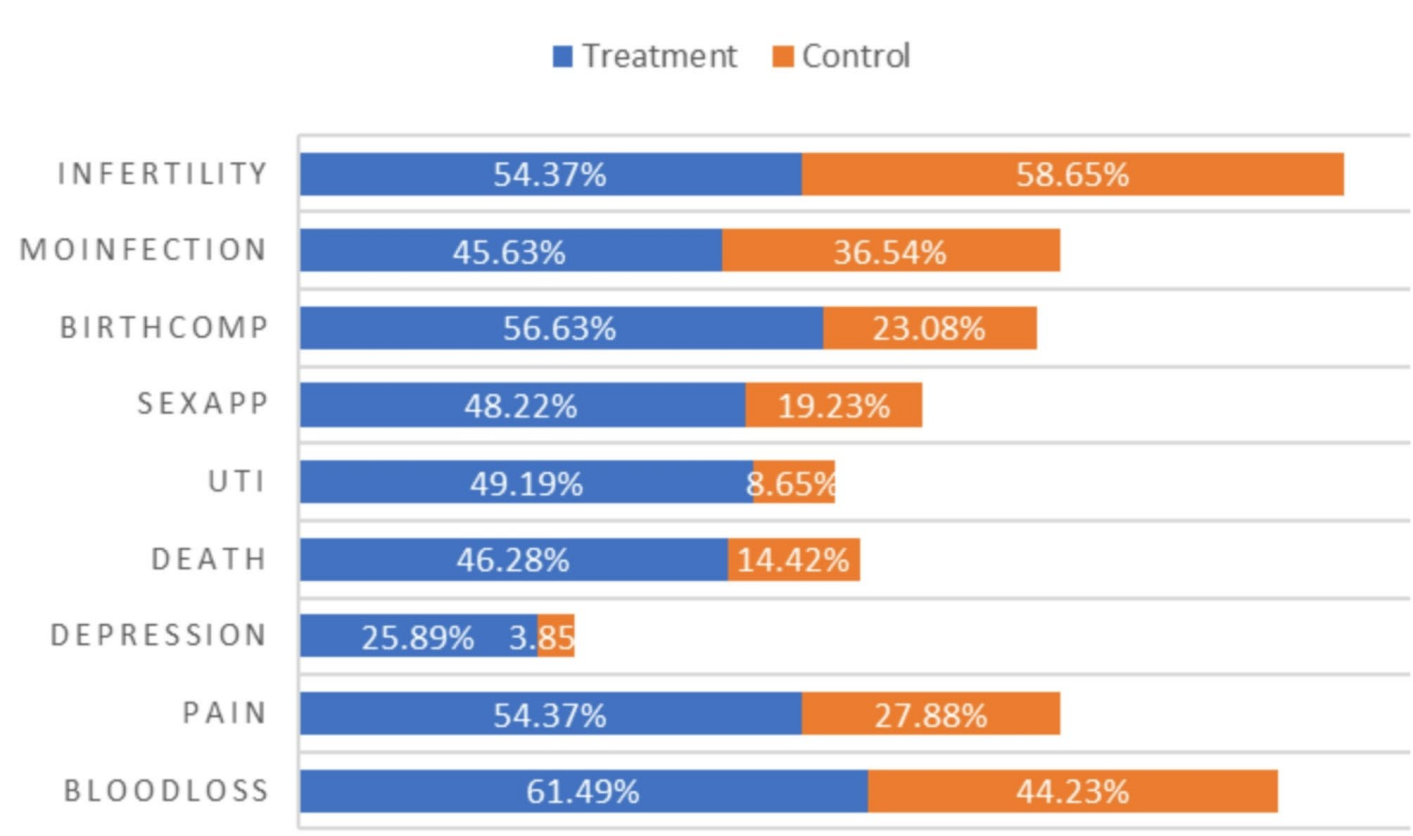
Knowledge on consequences of FGM/C

In terms of the consequences of FGM/C, Fig. 3 depicts the distribution of knowledge about the consequences of FGM/C among the study participants. The highest reported consequence was infertility, with 58.65% in the control group and 54.37% in the treatment group. The next most commonly known consequence was severe blood loss, this was more reported in the treatment group (44.23%) compared to the control group (27.88%). Other consequences noted were general pain 27.88% in the control group and 54.37% in the treatment group. Notably, only 25.89% of participants receiving treatment reported knowledge that FGM/C can lead to depression, with this figure being substantially lower in the control group at 3.85%.

### Knowledge of FGM/C laws

**Fig. 4:**
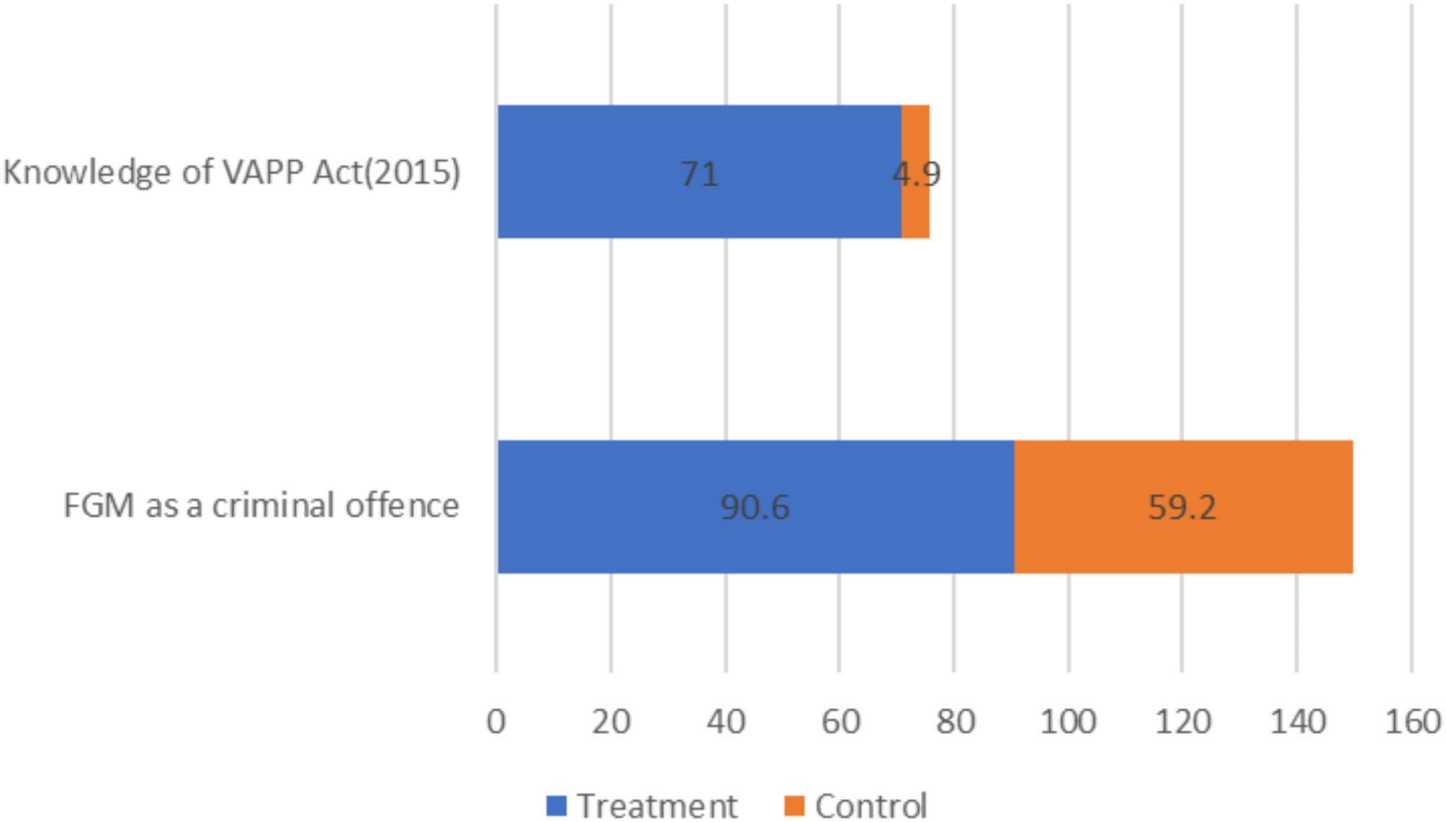
Knowledge of FGM/C Law

An assessment of the knowledge of FGM/C laws revealed that 90.6% and 59.2% of the treatment and control respondents respectively agreed that FGM/C is a criminal offense respectively. In addition, most (71.0%) of the treatment respondents knew the VAPP Act (2015) while only 4.9% of the control respondents (Fig. 4). This shows that most people are not well informed of the VAPP Act (2015) calling for increased sensitization.

However, a look at the differences between treatment and control groups, as shown in Table A2, we find significant differences between project participants and non-project participants. The signs are as expected. Overall, the significant mean differences for some covariates suggest that observed outcomes for non-project participants may not provide good counterfactuals for project participants. Estimation assuming random treatment assignment would therefore produce biased results thus calling for an alternative program evaluation technique. As such we used the IPW model.

### IPW model estimation results

If treatment assignment i.e., participation in the StopCut project was completely random, then a simple ordinary least square regression model or a comparison of the mean difference in the outcomes would suffice. However, since households could self-select themselves to participate in the StopCut project based on their level of education, social networks, or access to information, a random treatment assignment may not apply in this case. We, therefore, adopted the IPW regression model to address selectivity issues.

Before discussing the results, the underlying premises of IPW i.e., confoundedness and overlap must be met as shown in Figure A1 in the annex. The results depicted sufficient overlap although there are few propensity scores closer to either one or zero. This implies that the regions are too close to zero or one will not be within the common support. We also assessed the balance although we do not report them here. The primary IPW estimates are presented in Table 1.

**Table 1:** Inverse probability weighting model results

Variable	Knowledge of FGM/C Consequences	Knowledge of FGM/C as a Criminal offence	Knowledge of the Vapp Act	Willingness to Report FGM/C	Report index	Report FGM/C practiced by family members	Number of circumcised people known	Intention to practice FGM/C
Treatment	1.001*** (0.272)	0.021 (0.021)	0.533*** (0.043)	0.161** (0.053)	0.475** (0.233)	0.060* (0.031)	1.633** 0.746	0.082** (0.030)
Constant	2.955*** (0.248)	0.919 *** (0.016)	0.070** 0.033	0.719*** (0.045)	1.897*** (0.203)	0.856*** (0.028)	1.555*** (0.353)	0.042 (0.024)

Note: The effect estimates are reported as Beta-coefficients. Standard errors in parentheses. *** *p* < 0.01, **p < 0.05, *p < 0.1

The results show that participation in the StopCut project led to an increase in: knowledge of FGM/C consequences; knowledge of the VAPP Act (2015); willingness to report; reporting index; report of FGM/C practiced by family members; number of circumcised people known; and unexpectedly an increase in intention to practice FGM/C.

## Discussion

The results show evidence of the treatment effect of the StopCut interventions in agreement with the test of mean difference in Table A2. Despite the sensitivity to the choice of counterfactual, the direction and size of the program impacts may not be particularly sensitive to the inclusion of a broader set of covariates. The results confirmed that the impact of participation in the StopCut project was significant and positive on knowledge of FGM/C consequences, Knowledge of the VAPP Act (2015), Willingness to report FGM/C incidences, and reporting FGM/C practised by family members. It also led to an increase in the number of people known to practice circumcision. However, although unexpected the results revealed that participation in StopCut increased intention to practice FGM. This finding is unexpected as a reduction in intention to practice FGM/C was anticipated. Therefore, the findings suggest that the StopCut interventions are not effective in reversing the intention to practice FGM/C, necessitating additional qualitative research to contextualize the findings. One socially plausible explanation is that FGC/M is a deep-rooted sociocultural practice so addressing it might require a more comprehensive approach at all levels.

The findings from this study provide insights into the effectiveness of the StopCut project on the knowledge, beliefs, and practices related to FGM/C. The study indicates significant improvements in knowledge about the consequences of FGM/C, awareness of the Violence Against Persons Prohibition (VAPP) Act, willingness to report FGM/C, and a reduced intention to practice FGM/C. These outcomes reflect the intervention’s success in addressing several critical areas.

The significant improvement in knowledge regarding the consequences of FGM/C suggests that the StopCut intervention effectively communicated the severe health risks and broader implications of the practice. This aligns with previous studies, such as those by [30], which demonstrate that educational programs can effectively reduce the continuation of FGM/C by improving knowledge and attitudes. Enhancing awareness is crucial in shifting perceptions and fostering a deeper understanding of the harmful effects of FGM/C on women’s physical and psychological health.

The intervention’s success in raising awareness about the VAPP Act, 2015 is a pivotal achievement, as legal support is essential for the sustained prevention and eradication of FGM/C. This is consistent with the findings of [24], who emphasized the need for multifaceted, legislation-related interventions to be effective. Legal awareness serves as both a deterrent and a means of protecting women and girls who are at risk of FGM/C. The VAPP Act, 2015 provides a legal framework for the criminalization and persecution of the practice of FGM/C, a valuable tool for the protection of the rights of women and girls. By increasing the knowledge of participants on the VAPP Act 2015, the StopCut project improved their knowledge of the law against FGM/C and highlighted the practice as a violation of human rights. However, in further analysis, the results showed that though participants were aware of the VAPP Act 2015, there was no significant change in their recognition of FGM/C as a criminal offense. This could be inferred to mean that either participants did not fully understand the provisions of the law, or they still viewed FGM/C as not criminal even though there was a law against it. This shows a disconnect between people’s knowledge of the existence of the law, FGM/C being punishable by law, and their perception of the law being enforceable. This also indicates a possible gap in the intervention’s content or delivery of laws against FGM/C. Legal awareness campaigns must ensure that the legal ramifications of FGM/C are communicated and understood to be effective.

The significant increase in the willingness to report FGM/C among the intervention group is a positive outcome that indicates a shift towards proactive engagement in combating the practice. Reporting is a crucial step in the prevention and elimination of FGM/C, as it allows for legal and social measures to be taken against perpetrators. This finding is supported by the literature, which highlights the importance of community education and sensitization in encouraging reporting and breaking the silence surrounding FGM/C [43]. It also aligns with the literature suggesting that community education and sensitization can significantly impact attitudes [20, 30].

The reduced intention to practice FGM/C among the treatment group is one of the most significant outcomes of the StopCut intervention. Changing intentions and future behaviours is particularly challenging, given the deeply entrenched cultural norms surrounding FGM/C. The intervention’s success in this area suggests that it effectively challenged and began to alter these cultural norms. Studies, such as those by [19, 22], have similarly shown that community-led initiatives and social norm change campaigns can significantly reduce pro-FGM/C attitudes and behaviours.

The intervention significantly reduced the perception of FGM/C as beneficial among the treatment group, contrasting with the control group where this belief persisted. This highlights a critical challenge that deeply entrenched cultural beliefs are resistant to change, even in the face of interventions. The persistence of these beliefs in the control group underscores the necessity for sustained and multifaceted approaches to effectively challenge and shift cultural norms. The minimal support for the medicalization of FGM/C in the treatment group, contrasted with the substantial support in the control group, pointing to a nuanced understanding of FGM/C. The intervention appears to have effectively communicated the broader health risks and ethical concerns associated with medicalized FGM/C, beyond simply relocating the practice to a clinical setting.

## Conclusions

The findings indicate that the StopCut intervention effectively improved participants’ knowledge about FGM/C, legal frameworks, and reporting behaviour. The study thus highlights the need for a more comprehensive approach to challenging cultural beliefs and scaling up the interventions to other areas as well.

### Policy recommendations

The study recommends intensified efforts in community education and sensitization to dispel myths and misconceptions about FGM/C, emphasizing its harmful consequences, and promoting alternative rites of passage that do not involve cutting. This responsibility lies with state ministries of health and local government health departments, working in collaboration with community-based organizations.

Promoting all FGM/C reporting platforms is crucial to ensure community engagement. State-level law enforcement agencies, supported by the federal level, should lead efforts to publicize and operationalize these platforms effectively.

Despite existing laws prohibiting FGM/C in Nigeria, enforcement remains a significant challenge. The federal government should allocate resources for the prosecution of FGM/C cases, while state and local governments strengthen law enforcement mechanisms and establish monitoring systems.

Scaling up StopCut interventions to other areas and adapting them to diverse cultural contexts would further enhance their impact. State governments, in partnership with local governments and civil society organizations (CSOs), should champion this expansion.

The EndFGM Alliance and capacity-building workshops should also be expanded to reach a wider population. Federal and state governments, supported by international donors and non-governmental organizations (NGOs), should provide funding and logistical support for these initiatives.

Civil society organizations play a vital role in FGM/C prevention and response. Empowering them with the necessary resources, training, and support will enable them to effectively implement community-level interventions, monitor FGM/C cases, and provide support services to survivors. This effort should be coordinated by state governments with financial backing from federal agencies and international partners.

Targeted interventions should integrate men and boys in efforts to challenge harmful gender norms and promote their active involvement in advocating for the abandonment of FGM/C. Local government authorities, working with traditional leaders and youth organizations, should spearhead these efforts.

Furthermore, the issue of the medicalization of FGM/C must be addressed by raising awareness among healthcare providers about the harms of FGM/C, regardless of the performer, and enforcing strict regulations against the medicalization of the practice. State ministries of health and professional medical associations should take the lead in these efforts.

### Areas for further research

Future research should focus on exploring the underlying reasons for the continued practice of FGM/C despite interventions. Investigating the effectiveness of various approaches in different cultural and geographic settings will provide valuable insights. Longitudinal studies are essential to understanding the evolving nature of FGM/C and the impact of social, economic, and political changes. Effective collaboration and coordination among government agencies, civil society organizations, community leaders, and other stakeholders are crucial for achieving a collective impact. Research should identify best practices for joint planning, information sharing, and regular dialogue to enhance comprehensive efforts against FGM/C. Additionally, studies should examine the role of gender dynamics in perpetuating FGM/C and the effectiveness of advocacy efforts targeting men and boys. Continued investment in research and evaluation will be critical for identifying and implementing effective strategies to eliminate FGM/C.

### Limitations of the study

One limitation of the study is that although participants were randomly selected for the project, the sampling approach would probably yield some biased results which may need employing some instrumental variable methods for causal analysis. However, there was no suitable instrument (or instrumental variable) for causal analysis. Another limitation of the study is that the data collection tool captured mostly variables on knowledge with very few capturing attitudes and practices. However, despite the absence of a plausible instrument and limited variables on practices and attitudes towards FGM, the use of Inverse Probability Weighting (IPW) mitigated the likely selection bias between the groups, achieving comparability in observed covariates and improving the rigor of the causal analysis.

## Electronic supplementary material

Below is the link to the electronic supplementary material.

Supplementary Material 1

## Data Availability

Data used in the study will be availed on request.

## Footnotes

1 Medicalization of FGM refers to situations in which FGM is practiced by any category of health care provider, whether in public or private clinic, at home or elsewhere (UNFPA 2018). This is done to reduce harmful consequences of the practice.

## References

[1] WHO, Female genital mutilation, WHO. 2024. https://www.who.int/news-room/fact-sheets/detail/female-genital-mutilation. Accessed 6 Oct 2024.

[2] Reisel D, Creighton SM. Long term health consequences of female genital mutilation (FGM). Maturitas. 2015;80:48–51. 25466303 10.1016/j.maturitas.2014.10.009

[3] WHO. WHO guidelines on the management of health complications from female genital mutilation. 2016. 27359024

[4] Female. genital mutilation A global concern. 2024.

[5] Abdulcadir J, Catania L, Hindin MJ, Say L, Petignat P, Abdulcadir O. Female Genit Mutilation Obstet Gynecol. 2016;128:958–63. 10.1097/AOG.0000000000001686 27741194

[6] UNFPA. FGM/C in Nigeria: Telling Stories, Raising Awareness, Inspiring Change. 2019.

[7] Akweongo P, Jackson EF, Appiah-Yeboah S, Sakeah E, Phillips JF. It’s a Woman’s thing: gender roles sustaining the practice of female genital mutilation among the Kassena-Nankana of Northern Ghana. Reprod Health. 2021;18. 10.1186/s12978-021-01085-z PMC7923333 33648528

[8] Donohoe M. Female Genital Cutting: Epidemiology, Consequences, and Female Empowerment. 2006.

[9] Osifo DO, Evbuomwan I. Female Genital Mutilation among Edo People: The Complications and Pattern of Presentation at a Pediatric Surgery Unit, Benin City. 2009. 20687262

[10] Bjälkander O, Bangura L, Leigh B, Berggren V, Bergström S, Almroth L. Health complications of female genital mutilation in Sierra Leone. Int J Womens Health. 2012;4:321–31. 22870046 10.2147/IJWH.S32670 PMC3410700

[11] Kimani SJM-SN. Health impacts of female genital mutilation/cutting: A synthesis of the evidence. Evid End FGM/C Programme: Res Help Girls Women Thrive. 2016. 10.31899/rh8.1006.

[12] Berg RC, Taraldsen S, Said MA, Sørbye IK, Vangen S. Reasons for and experiences with surgical interventions for female genital mutilation/cutting (FGM/C): A systematic review. J Sex Med. 2017;14:977–90. 28666656 10.1016/j.jsxm.2017.05.016

[13] THE LAW AND FGM AN OVERVIEW. OF 28 AFRICAN COUNTRIES. 2018.

[14] Slanger T, Snow RC, Oronsaye F, Okonofua F, Wacker J. Female genital cutting in Southern urban and peri-urban Nigeria: Self-reported validity, social determinants and secular decline. Trop Med Int Health. 2002;7:91–100. 11851959 10.1046/j.1365-3156.2002.00829.x

[15] Ibrahim SB, And Ukaibe AG, Analysis of information sources, knowledge and attitude of gbongan residents (OSUN STATE). towards female genital mutilation. J Community Communication Res. 2023;8.

[16] Mandara MU. Female genital mutilation in Nigeria. Int J Gynecol Obstet. 2004;84:291–8. 10.1016/j.ijgo.2003.06.001 15001386

[17] Muthumbi, Jane, et al. Female genital mutilation: a literature review of the current status of legislation and policies in 27 African countries and Yemen. Afr J Reprod Health. 2015;19(3):32–40. https://www.ajol.info/index.php/ajrh/article/view/124907. 26897911

[18] Ekundayo R, Robinson S. An evaluation of Community-Based interventions used on the prevention of female genital mutilation in West African countries. Eur Sci J ESJ. 2019;15.

[19] Evans WD, Donahue C, Snider J, Bedri N, Elhussein TA, Elamin SA. The Saleema initiative in Sudan to abandon female genital mutilation: outcomes and dose response effects. PLoS ONE. 2019;14. 10.1371/journal.pone.0213380 PMC6413931 30861029

[20] Mwendwa P, Mutea N, Kaimuri MJ, De Brún A, Kroll T. Promote locally led initiatives to fight female genital mutilation/cutting (FGM/C) lessons from anti-FGM/C advocates in rural Kenya. Reprod Health. 2020;17. 10.1186/s12978-020-0884-5 PMC7048066 32111249

[21] Muluneh MD, Stulz V, Francis L, Agho K. Gender based violence against women in sub-saharan Africa: A systematic review and meta-analysis of cross-sectional studies. Int J Environ Res Public Health. 2020;17. 10.3390/ijerph17030903 PMC7037605 32024080

[22] Muhula S, Mveyange A, Oti SO, Bande M, Kayiaa H, Leshore C et al. The impact of community led alternative rite of passage on eradication of female genital mutilation/cutting in Kajiado County, Kenya: A quasi-experimental study. PLoS ONE. 2021;16 4 April 2021. 10.1371/journal.pone.0249662 PMC8081212 33909635

[23] Farouki L, El-Dirani Z, Abdulrahim S, Akl C, Akik C, McCall SJ. The global prevalence of female genital mutilation/cutting: A systematic review and meta-analysis of National, regional, facility, and school-based studies. PLoS Med. 2022;19. 10.1371/journal.pmed.1004061 PMC9436112 36048881

[24] Matanda DJ, Van Eekert N, Croce-Galis M, Gay J, Middelburg MJ, Hardee K. What interventions are effective to prevent or respond to female genital mutilation? A review of existing evidence from 2008–2020. PLOS Global Public Health. 2023;3. 10.1371/journal.pgph.0001855 PMC10187928 37192150

[25] Ahanonu EL, Victor O. Mothers’ perceptions of female genital mutilation. Health Educ Res. 2014;29:683–9. 24412809 10.1093/her/cyt118

[26] Mberu, Blessing U. Female genital mutilation/cutting in Nigeria: a scoping review, evidence to end FGM/C: research to help women thrive. New York: Population Council. 2017. 10.31899/rh7.1023.

[27] Joyce AM, Ejukonemu. Gender and sexuality: the myth of female genital mutilation. J Cult Religious Stud. 2021;9.

[28] Matanda, Dennis, Melanie Croce-Galis, Jill Gay, and Karen Hardee. Effectiveness of interventions designed to prevent or respond to female genital mutilation: a review of evidence. Nairobi: UNFPA, UNICEF, WHO, and Population Council Kenya. 2021. 10.31899/sbsr2021.1017.

[29] Magangi, M. G. Effectiveness of anti-female genital cutting interventions on the psychosocial wellbeing of the girl child in marani sub-county, Kisii County, Kenya (Doctoral dissertation, KISII UNIVERSITY). (2023).

[30] Khalil AI, Orabi AMA, Community-Based, Intervention. Impact of an educational program in exchanging knowledge, attitude, and practices of female genital mutilation (FGM). Health Care Curr Rev. 2017;05.

[31] Kandala NB, Nnanatu CC, Atilola G, Komba P, Mavatikua L, Moore Z et al. A Spatial analysis of the prevalence of female genital mutilation/cutting among 0–14-year-old girls in Kenya. Int J Environ Res Public Health. 2019;16. 10.3390/ijerph16214155 PMC6862646 31661902

[32] UNFPA-UNICEF. Comprehensive Approach to Accelerating the Elimination of Female Genital Mutilation. 2022.

[33] UNFPA-UNICEF. COVID-19 DISRUPTING SDG 5.3: ELIMINATING FEMALE GENITAL MUTILATION. 2020.

[34] UNFPA. Interim Technical Note Impact of the COVID-19 Pandemic on Family Planning and Ending Gender-based Violence, Female Genital Mutilation and Child Marriage. 2020.

[35] von Elm E, Altman DG, Egger M, Pocock SJ, Gøtzsche PC, Vandenbroucke JP. The strengthening the reporting of observational studies in epidemiology (STROBE) statement: guidelines for reporting observational studies. J Clin Epidemiol. 2008;61:344–9. 18313558 10.1016/j.jclinepi.2007.11.008

[36] Izudi J, Bajunirwe F, Cattamanchi A. Negative effects of undernutrition on sputum smear conversion and treatment success among retreatment cases in Uganda: A quasi-experimental study. J Clin Tuberc Other Mycobact Dis. 2024;35. 10.1016/j.jctube.2024.100422 PMC10907175 38434999

[37] Izudi J, Kiragga AN, Kalyesubula P, Okoboi S, Castelnuovo B. Effect of the COVID-19 pandemic restrictions on outcomes of HIV care among adults in Uganda. Med (United States). 2022;101:E30282. 10.1097/MD.0000000000030282 PMC10980429 36086721

[38] Dehejia, R. H., & Wahba, S. Propensity score-matching methods for nonexperimental causal studies. Rev Econ Stat. 2002;84(1):151–61. 10.1162/003465302317331982.

[39] Rosenbaum PR, Rubin DB. The central role of the propensity score in observational studies for causal effects. 1083.

[40] HECKMAN J. SMITH J. Evaluating the welfare State, Frisch centenar. Cambridge University Press; 1998.

[41] By A, Colin Cameron PKT. Micro econometrics: Methods and Applications. 2005.

[42] Wooldridge JM, Standard-Nutzungsbedingungen. Inverse probability weighted estimation for general missing data problems. 10.1920/wp.cem.2004.0504

[43] UNFPA. Sustaining the Momentum Performance Report UNFPA-UNICEF Joint Programme on the Elimination of Female Genital Mutilation: Accelerating Change. 2023.

