# Supplementary material for: Impact of the Stopcut project on the practice of Female Genital Mutilation/Cutting in Southwest Nigeria: a quasi-experimental study
Source: BMC Public Health. 2025 Feb 24;25:768. doi: 10.1186/s12889-025-21976-1 (PMC11852816; doi:10.1186/s12889-025-21976-1)
Supplement: Supplementary file 1 — Supplementary Material 1 [file 12889_2025_21976_MOESM1_ESM.docx]

**SUPPLEMENTARY MATERIALS**

**Annex**

**Table A1: Summary Statistics**

|  | Whole(n=413) | | | | Treatment (n=309) | | | | Control (n=103) | | | |
| --- | --- | --- | --- | --- | --- | --- | --- | --- | --- | --- | --- | --- |
|  | Mean | SD | Min | Max | Mean | SD | Min | Max | Mean | SD | Min | Max |
| **Dependent Variables** | | | | | | | | | | | | |
| Knowledge of the consequences of FGM | 3.903 | 2.522 | 0.000 | 9.000 | 4.421 | 2.557 | 0.000 | 9.000 | 2.365 | 1.643 | 0.000 | 7.000 |
| 1 if Know that FGM is criminal | 0.910 | 0.286 | 0.000 | 1.000 | 0.951 | 0.215 | 0.000 | 1.000 | 0.788 | 0.410 | 0.000 | 1.000 |
| 1 if Know Vapp Act | 0.547 | 0.498 | 0.000 | 1.000 | 0.715 | 0.452 | 0.000 | 1.000 | 0.048 | 0.215 | 0.000 | 1.000 |
| 1 if WTR FGM incidence | 0.831 | 0.376 | 0.000 | 1.000 | 0.926 | 0.263 | 0.000 | 1.000 | 0.548 | 0.500 | 0.000 | 1.000 |
| Reporting index | 2.559 | 2.397 | 1.000 | 9.000 | 2.599 | 2.188 | 1.000 | 9.000 | 2.442 | 2.942 | 1.000 | 9.000 |
| 1 if respondent willing to report FGM done by family | 0.884 | 0.321 | 0.000 | 1.000 | 0.939 | 0.241 | 0.000 | 1.000 | 0.721 | 0.451 | 0.000 | 1.000 |
| Number of circumcised people known | 3.271 | 12.199 | 0.000 | 200.000 | 3.579 | 13.745 | 0.000 | 200.000 | 2.356 | 5.402 | 0.000 | 30.000 |
| 1 if have intention to circumcise female children | 0.116 | 0.321 | 0.000 | 1.000 | 0.139 | 0.347 | 0.000 | 1.000 | 0.048 | 0.215 | 0.000 | 1.000 |
| **Independent Variables** | | | | | | | | | | | | |
| 1 if the Respondent is female | 0.511 | 0.500 | 0.000 | 1.000 | 0.521 | 0.500 | 0.000 | 1.000 | 0.481 | 0.502 | 0.000 | 1.000 |
| Age of respondent | 29.690 | 14.259 | 10.000 | 80.000 | 30.340 | 14.409 | 10.000 | 80.000 | 27.760 | 13.690 | 10.000 | 66.000 |
| 1 if Respondent has post-secondary education | 0.814 | 0.390 | 0.000 | 1.000 | 0.838 | 0.369 | 0.000 | 1.000 | 0.740 | 0.441 | 0.000 | 1.000 |
| 1 if the respondent is a Christian | 0.542 | 0.499 | 0.000 | 1.000 | 0.515 | 0.501 | 0.000 | 1.000 | 0.625 | 0.486 | 0.000 | 1.000 |
| Number of children | 1.387 | 1.715 | 0.000 | 5.000 | 1.511 | 1.781 | 0.000 | 5.000 | 1.019 | 1.448 | 0.000 | 4.000 |
| 1 if the respondent is married | 0.441 | 0.497 | 0.000 | 1.000 | 0.482 | 0.500 | 0.000 | 1.000 | 0.317 | 0.468 | 0.000 | 1.000 |
| 1 if the respondent knows FGM | 0.923 | 0.268 | 0.000 | 1.000 | 0.968 | 0.177 | 0.000 | 1.000 | 0.788 | 0.410 | 0.000 | 1.000 |
| 1 if know the definition of FGM | 0.719 | 0.450 | 0.000 | 1.000 | 0.799 | 0.401 | 0.000 | 1.000 | 0.481 | 0.502 | 0.000 | 1.000 |
| 1 if FGM has benefit | 0.201 | 0.401 | 0.000 | 1.000 | 0.094 | 0.292 | 0.000 | 1.000 | 0.519 | 0.502 | 0.000 | 1.000 |
| 1 if FGM is good if done by doctors | 0.167 | 0.373 | 0.000 | 1.000 | 0.087 | 0.283 | 0.000 | 1.000 | 0.404 | 0.493 | 0.000 | 1.000 |
| 1 if Employed | 0.591 | 0.492 | 0.000 | 1.000 | 0.625 | 0.485 | 0.000 | 1.000 | 0.490 | 0.502 | 0.000 | 1.000 |
| Monthly income | 32217.915 | 84358.771 | 0.000 | 1500000.000 | 33712.084 | 94581.971 | 0.000 | 1500000.000 | 27778.510 | 41092.773 | 0.000 | 200000.000 |

**Table A2: Test of Mean difference**

| Variable | Treatment | | Controls | | Mean Difference | |
| --- | --- | --- | --- | --- | --- | --- |
|  | Mean | s.e | Mean | s.e | Mean | s.e |
| **Dependent Variables** |  |  |  |  |  |  |
| Knowledge of the consequences of FGM | 4.421 | 0.145 | 2.365 | 0.161 | -2.055*** | 0.267 |
| 1 if Know that FGM is criminal | 0.951 | 0.012 | 0.788 | 0.040 | -0.163*** | 0.031 |
| 1 if Know Vapp Act | 0.715 | 0.026 | 0.048 | 0.021 | -0.667*** | 0.046 |
| 1 if WTR FGM incidence | 0.926 | 0.015 | 0.548 | 0.049 | -0.377*** | 0.038 |
| Reporting index | 2.599 | 0.124 | 2.442 | 0.288 | -0.156 | 0.272 |
| 1 if respondent willing to report FGM done by family | 0.939 | 0.013 | 0.721 | 0.044 | -0.217*** | 0.035 |
| Number of circumcised people known | 3.579 | 0.782 | 2.356 | 0.530 | -1.224 | 1.383 |
| 1 if have intention to circumcise female children | 0.139 | 0.020 | 0.048 | 0.021 | -0.091*** | 0.036 |
| **Independent Variables** |  |  |  |  |  |  |
| 1 if the Respondent is female | 0.521 | 0.028 | 0.481 | 0.049 | -0.040 | 0.056 |
| Age of respondent | 30.339 | 0.820 | 27.760 | 1.342 | -2.580 | 1.613 |
| 1 if Respondent has post-secondary education | 0.838 | 0.021 | 0.740 | 0.043 | -0.098** | 0.043 |
| 1 if the respondent is a Christian | 0.515 | 0.028 | 0.625 | 0.048 | 0.110** | 0.056 |
| Number of children | 1.511 | 0.101 | 1.019 | 0.142 | -0.492*** | 0.193 |
| 1 if the respondent is married | 0.482 | 0.028 | 0.317 | 0.046 | -0.165*** | 0.056 |
| 1 if the respondent knows FGM | 0.968 | 0.0100 | 0.788 | 0.040 | -0.179*** | 0.029 |
| 1 if know the definition of FGM | 0.799 | 0.023 | 0.481 | 0.049 | -0.319*** | 0.048 |
| 1 if FGM has benefit | 0.094 | 0.017 | 0.519 | 0.049 | 0.425*** | 0.040 |
| 1 if FGM is good if done by doctors | 0.087 | 0.016 | 0.404 | 0.048 | 0.316*** | 0.039 |
| 1 if Employed | 0.625 | 0.028 | 0.490 | 0.049 | -0.134** | 0.055 |
| Monthly Income | 33712.08 | 5380.58 | 27778.51 | 4029.48 | -5933.58 | 9570.49 |

**Figure A1: Kernel Density before and after matching**


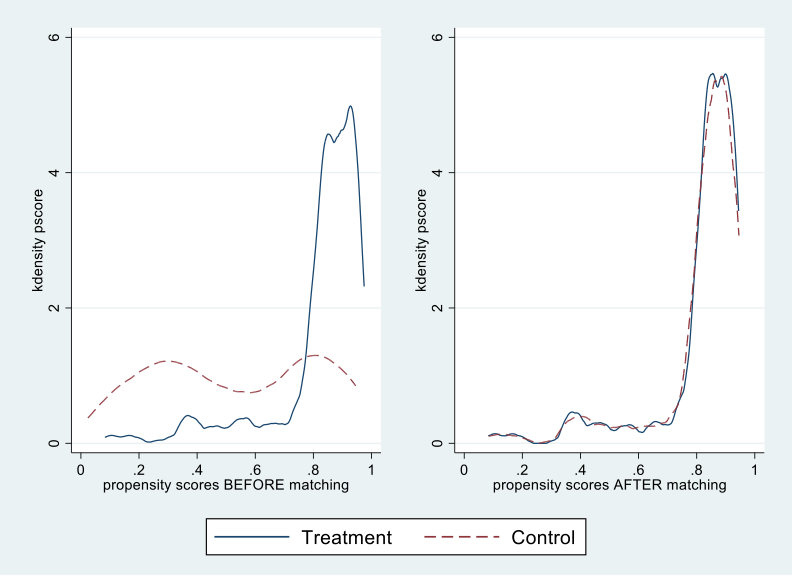


**Theory of Change**


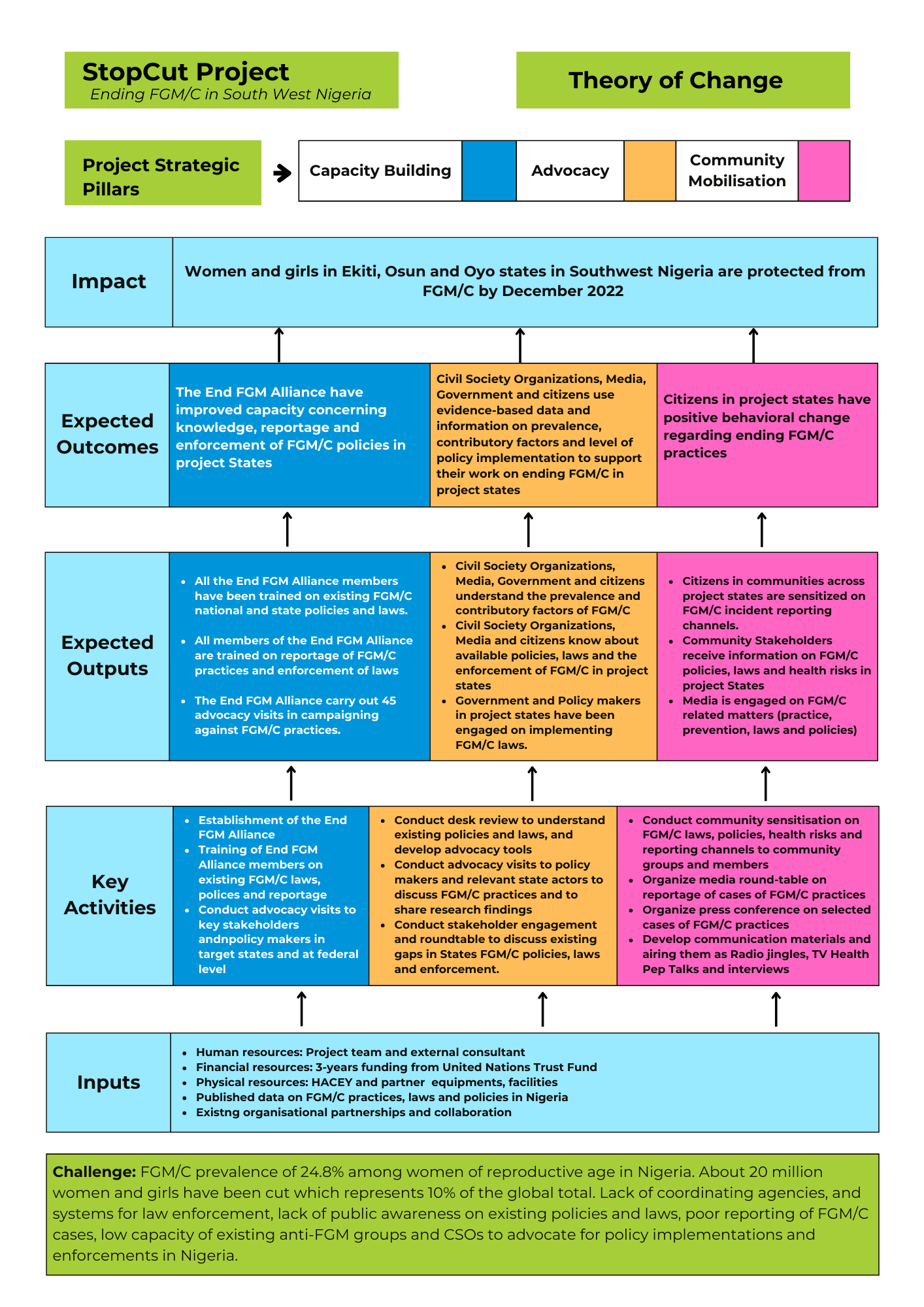


**ETHICA APPROVAL**


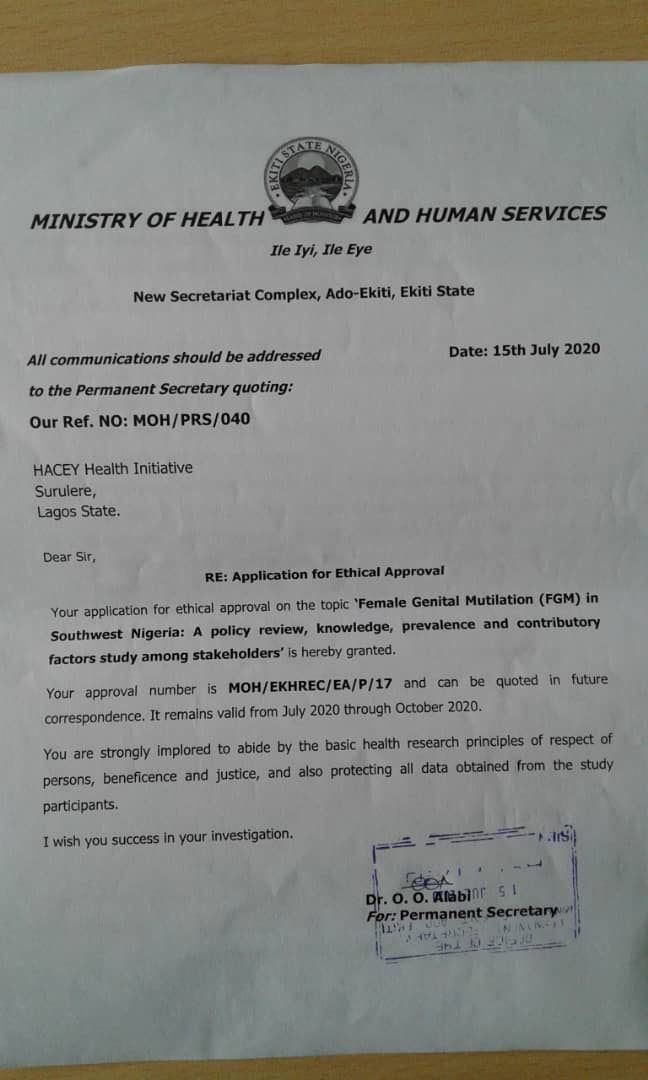

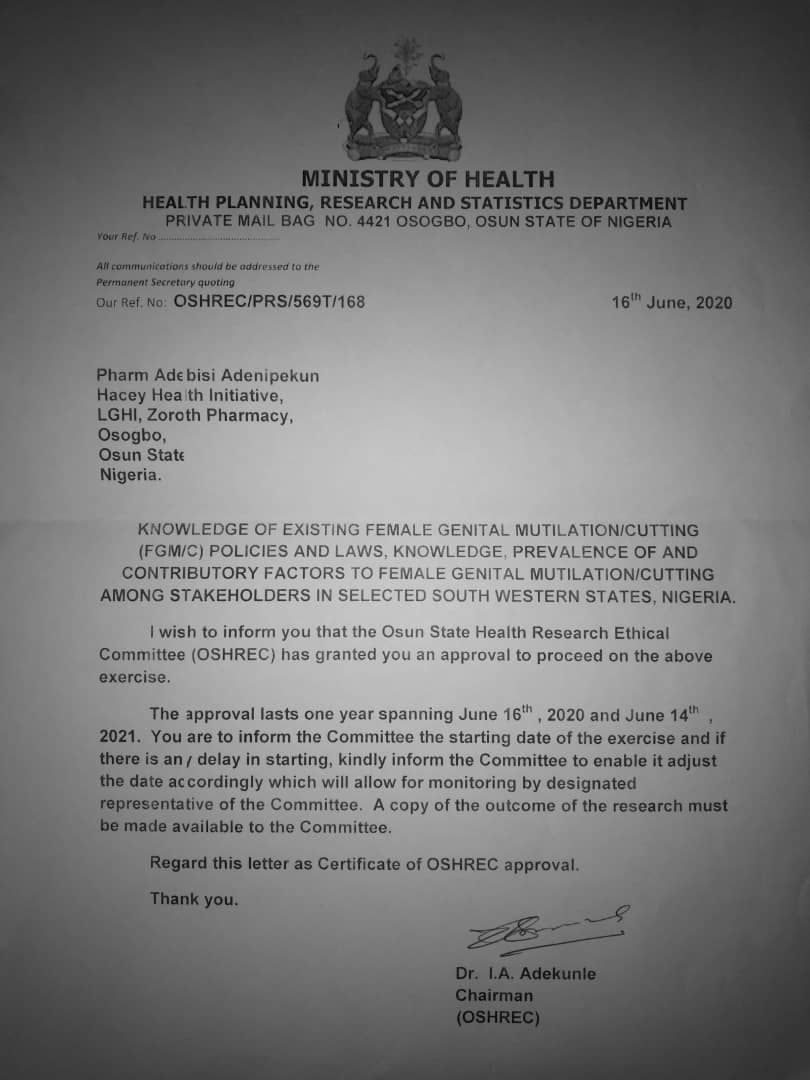

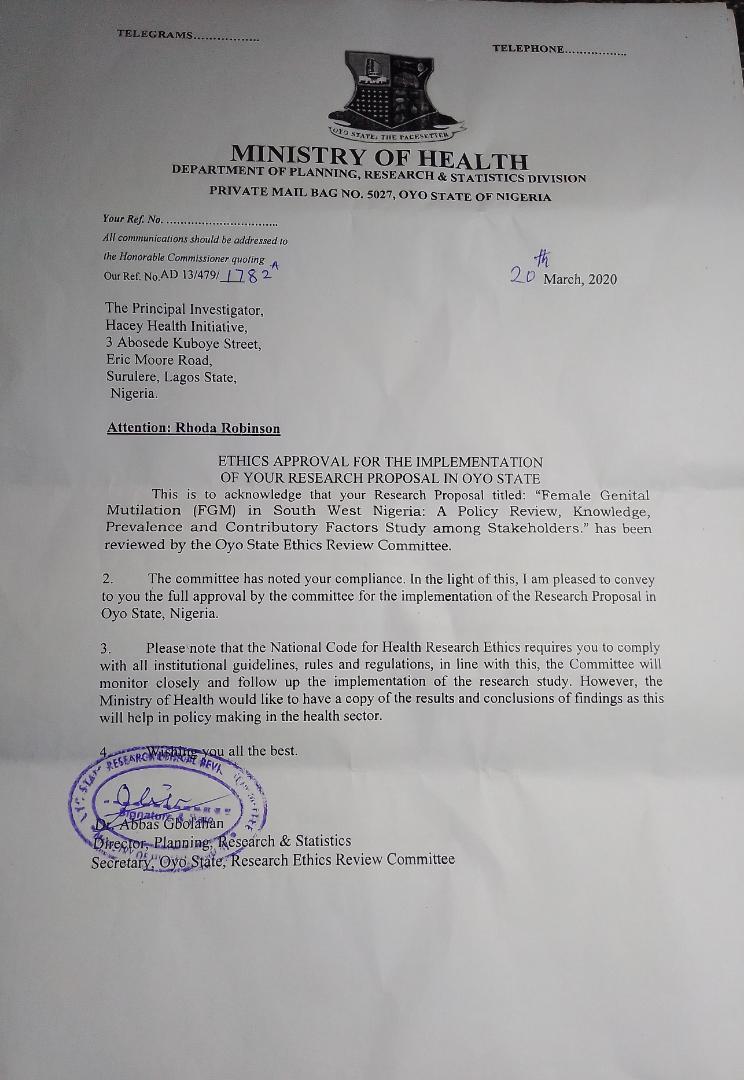


**INFORMED CONSENT**

**INFORMED CONSENT LETTER FOR COMMUNITY MEMBERS IN SOUTH-WEST NIGERIA**
**STUDY TITLE:** **Impact of the StopCut Project on the Practice of Female Genital Mutilation/Cutting (FGM/C) in South-West Nigeria**

**Introduction/Purpose:**
The HACEY Health Initiative is conducting a research study aimed at assessing the impact of the **StopCut Project**, which seeks to reduce the practice of Female Genital Mutilation/Cutting (FGM/C) in South-West Nigeria. The purpose of this study is to understand community members’ perspectives on FGM/C and the effectiveness of the StopCut Project in reducing the practice. We kindly request your participation by completing a questionnaire that will help us gather information about your experiences and opinions on this subject.

**Study Procedure:**

If you decide to participate, you will be asked a series of questions related to FGM/C and the impact of the StopCut Project in your community. The questionnaire will take approximately 15-20 minutes to complete. You are encouraged to answer all questions, but you may skip any question that you are uncomfortable answering.

**Compensation:**
There is no financial compensation for participating in this study. However, your participation is invaluable as it will help shape future efforts to eliminate FGM/C in your community and across the region.

**Confidentiality:**
Your responses are completely anonymous and will be treated with the utmost confidentiality. No personally identifying information will be linked to your responses. A unique registration number will be assigned to your questionnaire, and only the research team will have access to the data for analysis purposes.

**Potential Risks:**

There are no known risks associated with participating in this study. However, if any question makes you feel uncomfortable, you are free to skip that question or stop participating at any time.

**Voluntary Participation/Withdrawal from the Study:**

Your participation in this study is entirely voluntary. You may choose to withdraw from the study at any time, and this decision will not affect you in any way.

**If you are willing to participate, please sign below:**

**Signature_______________________**

Thank you for taking the time to participate in this important research.

**QUESTIONNAIRE**

**IMPACT OF THE STOPCUT PROJECT ON THE PRACTICE OF FEMALE GENITAL MUTILATION/CUTTING IN SOUTHWEST, NIGERIA**

**Dear respondent,**

My name is __________ I am a data collector representing HACEY Health Initiative, Lagos, Nigieria. We are currently carrying out a research study to determine the impact of the StopCut project on the practice of Female Genital Mutilation/Cutting in South-West, Nigeria. The questionnaire below is designed to get your response and view about this research, and I am looking forward to positive feedback.

Your participation is voluntary, and your responses will be kept confidential. We greatly value your input, as it will contribute to shaping future efforts aimed at eliminating FGM in our region. Thank you for taking the time to share your views

**Section A: Socio-Demographic Characteristics**

1. Gender: Male ( ) Female ( )
2. State:__________________
3. Age: __________________
4. Highest Educational Level Attained: Primary ( ) Secondary ( ) Tertiary ( ) Vocational ( ) No Education ( )
5. Marital Status: Cureently married ( ) Single ( ) Single Parent ( ) Divorced ( ) Widow ( )
6. Number of children given birth to_________
7. Ethnic Group: Youruba ( ) Igbo ( ) Hausa ( ) Others ______
8. Religion: Christainity ( ) Islam ( ) Traditional ( )
9. Employment status : Fully employed ( ) Self employed ( ) Volunteer ( ) Not employed ( )
10. Average monthly income: __________

**Section B: Awareness of Female Genital Mutilation**/**Cutting**

1. Have you ever heard about Female Genital Mutilation/Cutting? Yes ( ) No ( )
2. If yes where did you hear about FGM/C? community meeting ( ) school ( ) Friends ( ) Family members ( ) Church ( ) Mosque ( ) Mothers ( ) Social gatherings ( ) Women Group Meetings ( ) Others ____

**Section C: Knowledge of** **Female Genital Mutilation**

1. Can you define FGM/C? Yes ( ) No ( )
2. FGM is not wrong when a doctor or health worker does it: Yes ( ) No ( )
3. How many classes of FGM/C exist? ________
4. FGM/C has some benefits: Yes ( ) No ( )
5. If your response to the previous question was yes please can you state the benefit?

- Loss blood Yes ( ) No ( )
- Pain Yes ( ) No ( )
- Depression Yes ( ) No ( )
- Death Yes ( ) No ( )
- Urinary Tract Infection Yes ( ) No ( )
- Lack of sexual appetite Yes ( ) No ( )
- Birth Complications Yes ( ) No ( )
- Others Yes ( ) No ( )

**Section D: Perception of** **Female Genital Mutilation/Cutting**

1. Uncircumcised women get more infections: Agree ( ) Neutral ( ) Disagree ( )
2. FGM/C can cause infertilty: Agree ( ) Neutral ( ) Disagree ( )
3. FGM/C is legal in Nigeria: Agree ( ) Neutral ( ) Disagree ( )
4. FGM/C is not dangerous: Agree ( ) Neutral ( ) Disagree ( )
5. FGM/C does not cause painful sexual intercourse: Agree ( ) Neutral ( ) Disagree ( )
6. FGM/C can cause severe bleeding: Agree ( ) Neutral ( ) Disagree ( )
7. Women who have been mutilated are more likely to suffer from urinary problem: Agree ( ) Neutral ( ) Disagree ( )
8. FGM cannot cause cancer: Agree ( ) Neutral ( ) Disagree ( )
9. Being circumcised makes no difference during childbirth: Agree ( ) Neutral ( ) Disagree ( )
10. If the clitoris is removed it will grow large, like a penis: Agree ( ) Neutral ( ) Disagree ( )
11. if the clitoris is not removed, the baby will die during delivery: Agree ( ) Neutral ( ) Disagree ( )
12. Circumcised women are less likely to catch STIs: Agree ( ) Neutral ( ) Disagree ( )

**Section E: Female Genital Mutilation/Cutting Laws and Policies**

1. Do you know that FGM/C is a criminal offense? Yes ( ) No ( )
2. Are there state laws that prohibit FGM/C? Yes ( ) No ( )
3. Have you ever heard about the VAPP Act? Yes ( ) No ( )
4. Do you know what the VAPPAct is? Yes ( ) No ( )
5. Has the VAPPAct been passed in your state? Yes ( ) No ( )
6. How many years in prison will someone who practice FGM/C spend in jail if caught? _____
7. According to the VAPPAct the fine for committing FGM/C is? ______
8. Supporting FGM/C practice without directly carrying out the act attracts how many years in prison? ________

**Section F: Reporting of Female Genital Mutilation/Cutting**

1. Will you report any incidence of FGM/C cases in your community? Yes ( ) No ( )
2. Where should FGM/C cases be reported to:

- Nigeria Police Force: Yes ( ) No ( )
- Community association: Yes ( ) No ( )
- Nigerian security and civil defense: Yes ( ) No ( )
- Amotekun security Yes ( ) No ( )
- Market women association Yes ( ) No ( )
- Schoolauthorities: Yes ( ) No ( )
- Church: Yes ( ) No ( )
- Mosque: Yes ( ) No ( )
- Others__________

1. Do you know that you can report FGM/C cases via phone call for free? Yes ( ) No ( )
2. Do you think FGM/C practice should be continued? Yes ( ) No ( )
3. Will you report cases of FGM/C in your family or community to security operative?

Yes ( ) No ( )

**Section G: Practice of Female Genital Mutilation/Cutting**

1. Were you Circumcised? Yes ( ) No ( )
2. Do you know people who were circumcised? Yes ( ) No ( )
3. How many do you know? __________
4. Are you aware of any in the last year? Yes ( ) No ( )
5. If yes how may people? ________
6. Will you practice FGM/C because of:

- Culture: Yes ( ) No ( )
- Religion: Yes ( ) No ( )
- Prevent promiscuity: Yes ( ) No ( )

1. Do you have the intention to circumcise female children? Yes ( ) No ( )
2. Will you recommend FGM/C to family and friends? Yes ( ) No ( )
